# Management and outcomes of chemotherapy-associated pneumoperitoneum in cancer patients: a scoping review protocol

**DOI:** 10.1097/SP9.0000000000000007

**Published:** 2023-09-18

**Authors:** Renee M. Maina, Caroline A. Rader, Jeevan Kypa, Hilary M. Jasmin, Constantine Asahngwa, Clarisse S. Muenyi, Denis A. Foretia

**Affiliations:** a Department of Surgery; b College of Medicine; c Health Science Library; d Center for Multicultural and Global Health; e Global Surgery Institute, University of Tennessee Health Science Center, Memphis, Tennessee, USA; f Division of Health Policy and Research, Nkafu Policy Institute, Yaoundé, Cameroon

**Keywords:** chemotherapeutic agent, chemotherapy, pneumoperitoneum, scoping review protocol, surgery

## Abstract

**Introduction:**

Pneumoperitoneum – free air within the peritoneal cavity – is often the result of bowel perforation, though other causes include residual postprocedural or postoperative air and barotrauma. In non-cancer patients, operative intervention is often required. Cancer patients, on the other hand, present a unique set of challenges as they usually have elevated risk of pneumoperitoneum from local radiation therapy, frequent endoscopic procedures, and tumor invasion. Factors such as malnutrition, neutropenia, chemotherapy, and steroid use make emergent surgery tenuous in cancer patients. There is a paucity of published literature on the management of pneumoperitoneum in patients actively undergoing chemotherapy. The main objective of this scoping review is to assess the presentation, management, and subsequent outcomes of this unique patient population.

**Materials and Methods:**

The authors will utilize the framework for performing scoping reviews as outlined by Arksey and O’Malley. They will perform the search for articles in three electronic databases (i.e. SCOPUS, PubMed, Embase) and relevant gray literature. Only articles available in English and published between 1999 and 2022 will be included. Inclusion criteria will be a known diagnosis of cancer, chemotherapy within 6 months of presentation, and imaging confirmation of pneumoperitoneum. Exclusion criteria will be cancer diagnosis at the time of presentation, perforation secondary to cancer itself, and chemotherapy greater than 6 months prior to presentation. A tailored extraction frame to extract relevant information from published articles that meet our inclusion criteria. The data using both descriptive statistics and thematic analysis of the main study questions.

**Ethics and Dissemination:**

Since the authors will not be collecting primary data, formal ethical approval is not required. They study findings will be disseminated through abstracts, conference presentations, and peer-reviewed publications.

## Introduction

Highlights

- Pneumoperitoneum is a surgical emergency that usually warrants exploratory laparotomy to rule out perforated viscus.
- Its management in cancer patients on chemotherapy remains poorly defined.
- There is a paucity of published literature on the management of pneumoperitoneum in patients actively undergoing chemotherapy.
- This scoping review evaluates the presentation, management, and outcomes in this unique patient population.

Pneumoperitoneum, defined as the presence of air in the peritoneal cavity, is often a surgical emergency that warrants an exploratory laparotomy to rule out perforated viscous. In fact, ~85–90% of cases of pneumoperitoneum are secondary to bowel perforation^1,2^. The other 10–15% of cases of pneumoperitoneum are caused by barotrauma, gynecological insufflation, and retained postoperative or postprocedural air^3^.

The gold standard for diagnosis of pneumoperitoneum is radiography. On plain film radiography, the presence of pneumoperitoneum is confirmed by a characteristic radiolucency below the diaphragm on chest radiograph or in a superiorly dependent location on abdominal radiograph^3,4^. Although plain radiography is typically the first study of choice when pneumoperitoneum is suspected, computed tomography (CT) is more sensitive in detecting not only intraperitoneal free air but also extraluminal air along the entire gastrointestinal tract^5,6^.

In non-cancer patients, the presence of pneumoperitoneum usually warrants emergent operative management. Cancer patients present a peculiar challenge with elevated risk of pneumoperitoneum as a result of frequent endoscopic procedures, radiotherapy, and local tumor invasion^1^. Patient-specific factors such as malnutrition and neutropenia make emergent surgery tenuous in cancer patients.

Additionally, emergent surgery on cancer patients presents an operative dilemma as these patients typically are immunosuppressed and/or undergoing active systemic chemotherapy, which increases postoperative morbidity^7^. In the case of patients undergoing neoadjuvant chemotherapy, there is the risk that emergent surgery may negatively impact the ability to obtain a complete (R0) resection for cure during the planned oncological surgery.

There is a paucity of published literature on the management of pneumoperitoneum in patients actively undergoing chemotherapy. We plan to conduct a scoping review on the presentation, management, and subsequent outcomes in this patient population.

## Aims of the scoping review

A preliminary search of the Cochrane Database of Systematic Reviews, CINAHL, and PROSPERO revealed no active, previous, or forthcoming scoping reviews on our proposed topic. The fundamental goal of our study is to identify the initial presentation, diagnosis, management, and ultimate outcomes of cancer patients who present with pneumoperitoneum that is caused by ongoing chemotherapy.

## Methodology

This scoping review will be guided by the five-step methodological framework proposed by Arksey and O’Malley^8,9^. This involves (i) identifying the research question; (ii) identifying relevant studies; (iii) selecting eligible studies; (iv) charting the data; and (v) collating and summarizing the results. We chose this framework because it ensures that a clearly delineated methodological and transparent process is adhered to when examining the extent, range, and nature of research activities, including identifying research gaps.

**Figure 1:**
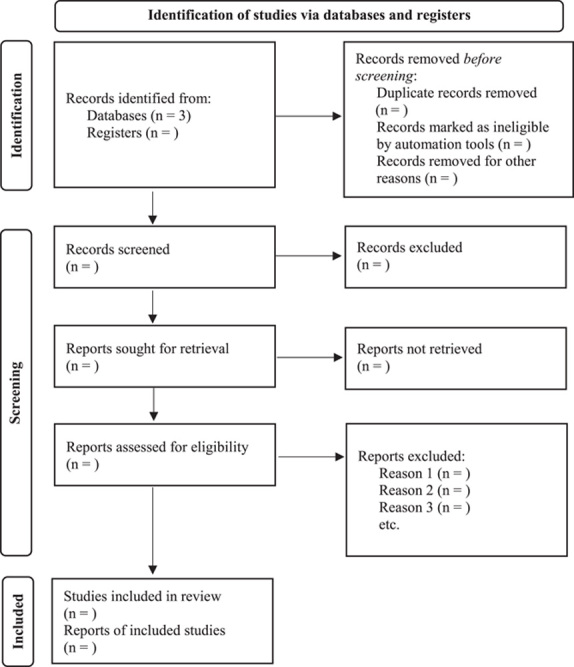
PRISMA (Preferred Reporting Item for Systematic Reviews and Meta-Analyses) flow diagram for proposed scoping review.

The Preferred Reporting Item for Systematic Reviews and Meta-Analyses extension for Scoping Reviews (PRISMA-ScR) will be used throughout the review process to guide the screening and reporting (Fig. 1)^10^. A scoping review is more appropriate than a systematic review as the study will include all cancers, all age groups, and all chemotherapeutic regimens to have a broad sense of factors predisposing to pneumoperitoneum in this patient population.

### Identifying the research question

This scoping review will use the iterative process to look at the concept, target population, and outcomes as described by Arksey and O’Malley. The concept driving the study is identifying a gap in the described healthcare management guidelines. The target population is patients with a known diagnosis of cancer who are actively undergoing chemotherapy. The outcomes will be delineating the optimum management for this patient population and developing a framework that can be used to guide a treatment algorithm.

### Identifying relevant studies

All team members, including the librarian, collaboratively developed and agreed upon the search strategy, and the inclusion and exclusion criteria were refined and agreed upon. A systematic search will be conducted for published and unpublished (gray) literature using a combination of keywords and Boolean operators ‘AND/OR.’

Three databases will be exhaustively searched: MEDLINE/PubMed, Embase, and Scopus. Only studies available in English will be considered. Search results will be exported to Endnote for data management and to remove duplicates. After removing duplicates, the data will then be exported to Rayyan, a free web tool designed to help researchers speed up the screening and selection of articles while working on systematic reviews and scoping reviews for screening. The screening process will be as follows:

#### Inclusion criteria

Articles published between 1999 and 2022 will be considered. Articles that are case reports and case series with clearly detailed extraction data will be included. Patient criteria for inclusion will be a known diagnosis of cancer, chemotherapy within 6 months of presentation, abdominal symptoms upon presentation, and imaging confirmation of pneumoperitoneum.

#### Exclusion criteria

Articles that are reviewed (retrospective or prospective) without specific patient details will be excluded. Patient exclusion criteria will be a new cancer diagnosis at the time of presentation, perforation secondary to the cancer itself, and chemotherapy greater than 6 months prior to presentation.

### Study procedure and selection of relevant studies

The titles and abstracts of all the articles exported to Rayyan will be double-blinded and screened by three independent reviewers (R.M.M., C.A.R., and J.K.) to select studies related to our population, intervention, comparators, and outcomes (PICO) based on the inclusion and exclusion criteria. Double screening of titles/abstracts will be checked independently by the reviewers (R.M.M., C.A.R., and J.K.) after unblinding.

Unresolved conflicts will be resolved through discussion, and all conflicting articles will be arbitrated by a fourth reviewer (D.A.F.). The second stage will involve full-text screening, which will be done by R.M.M., C.A.R., and J.K., each working independently. An attempt will be made to contact the authors of the articles without full text, while studies with multiple publications will have the latest of such publications retained.

### Data extraction

The data extraction form in Table 1 will be used to collect relevant information from all included studies.

**Table 1:** Data extraction form.

Specific focus	Data to be extracted
Demographic information	Age Sex
Oncologic information	Type of cancer Chemotherapy regimen Time between last chemotherapy session and presentation
Presentation and diagnosis	Presence or absence of abdominal symptoms Diagnostic imaging performed (plain film vs. computed tomography)
Intervention	Surgical management vs. nonsurgical management Site of perforation if surgically managed
Patient outcomes	Perioperative mortality 30-day mortality (if available) Perioperative complications (if available)

### Collating and summarizing the results

The overarching aim of our scoping review is to collect existing evidence on the management and subsequent outcomes of cancer patients presenting with chemotherapy-associated pneumoperitoneum. The data extracted will be analyzed using descriptive statistics and thematic analyses. The results will be summarized into key themes to identify current challenges and gaps in research.

### Data presentation

Our extracted data will be displayed in diagrammatic or tabular format to serve the goal of the review. A narrative summary will accompany the diagrammatic or tabular findings in the results.

## Ethics and Dissemination

Ethical approval is not required for this scoping review as data will be gathered by reviewing the current literature. Results of our study will be disseminated through abstracts, conference presentations, and peer-reviewed journal publications. Should we amend this protocol after publication, the date of amendment and specific changes will be communicated in addition to the rationale for the change.

## Patient and public involvement

There is no patient or public involvement in the design of this study.

## Discussion

Pneumoperitoneum in patients undergoing chemotherapy poses serious management challenges that often require a multidisciplinary team approach. Our scoping review, it is hoped, will help identify risk factors to be aware of and propose a management algorithm and strategies to reduce morbidity and mortality. Our study will provide insights on when patients on chemotherapy for solid and hematologic malignancies are likely to present with pneumoperitoneum, likely agents involved, and in patients managed operatively, the common sites of perforation. Importantly, our scoping review will provide directives for a future systematic review.

## Limitations

A limitation of this study is that it encompasses articles over a 23-year period (1999–2022). Although it excludes earlier articles, this period was chosen to capture more recent trends in the management of this patient population and thus aid in the formulation of current and practical patient management guidelines. Additionally, our scoping review will only look at data and articles published in English.

## Ethical approval

Ethical approval is not required for this scoping review as it analyses publicly available data.

## Consent

Not indicated.

## Sources of funding

This research did not receive any specific grant from funding agencies in the public, commercial, or not-for-profit sectors.

## Author contribution

R.M.M., C.A., and D.A.F.: conceptualization; R.M.M., C.A.R., J.K., H.M.J., and D.A.F.: data curation; R.M.M., C.A.R., J.K., and D.A.F.: formal analysis; R.M.M., C.A.R., J.K., H.M.J., and D.A.F.: investigation; R.M.M., C.A., H.M.J., and D.A.F.: methodology; D.A.F.: project administration; D.A.: supervision; R.M.M. and D.A.F.: writing – original draft; R.M.M., C.S.M., and D.A.F.: writing – review and editing.

## Conflicts of interest disclosure

There are no conflicts of interest.

## Research registration unique identifying number (UIN)

Not applicable.

## Guarantor

Denis A. Foretia, MD, MPH, MBA, FACS, Division of Health Policy and Research, Nkafu Policy Institute, The Denis & Lenora Foretia Foundation, Cameroon; E-mail: dforetia@foretiafoundation.org

## References

[1] Badgwell B Feig BW Ross MI. Pneumoperitoneum in the cancer patient. Ann Surg Oncol 2007;14:3141–3147. 17680315 10.1245/s10434-007-9510-9

[2] Cho KC Baker SR. Extraluminal air. Diagnosis and significance. Radiol Clin North Am 1994;32:829–844. 8084998

[3] Mularski RA Sippel JM Osborne ML. Pneumoperitoneum: a review of nonsurgical causes. Crit Care Med 2000;28:2638–2644. 10921609 10.1097/00003246-200007000-00078

[4] Singh JP Steward MJ Booth TC. Evolution of imaging for abdominal perforation. Ann R Coll Surg Engl 2010;92:182–188. 20412668 10.1308/003588410X12664192075251 PMC3080072

[5] Stapakis JC Thickman D. Diagnosis of pneumoperitoneum: abdominal CT vs. upright chest film. J Comput Assist Tomogr 1992;16:713–716. 1522261

[6] Yeung KW Chang MS Hsiao CP. CT evaluation of gastrointestinal tract perforation. Clin Imaging 2004;28:329–333. 15471663 10.1016/S0899-7071(03)00204-3

[7] Cauley CE Panizales MT Reznor G. Outcomes after emergency abdominal surgery in patients with advanced cancer: opportunities to reduce complications and improve palliative care. J Trauma Acute Care Surg 2015;79:399–406. 26307872 10.1097/TA.0000000000000764 PMC4552078

[8] Arksey H O’Malley L. Scoping studies: towards a methodological framework. Int J Soc Res Methodol 2005;8:19–32.

[9] Page MJ McKenzie JE Bossuyt PM. The PRISMA 2020 statement: an updated guideline for reporting systematic reviews. Syst Rev 2021;10:89. 33781348 10.1186/s13643-021-01626-4 PMC8008539

[10] Tricco AC Lillie E Zarin W. PRISMA extension for scoping reviews (PRISMA-ScR): checklist and explanation. Ann Intern Med 2018;169:467–473. 30178033 10.7326/M18-0850

